# Treatment of Priapism with Automated Red Cell Exchange and Hyperbaric Oxygen in an 11-year-old Patient with Sickle Cell Disease

**DOI:** 10.5505/tjh.2012.78553

**Published:** 2012-10-05

**Authors:** Fatih Mehmet Azık, Avni Atay, Ahmet Emin Kürekçi, Hakan Ay, Yusuf Kibar, Okan Özcan

**Affiliations:** 1 Gülhane Military Medical Academy, Department of Pediatric Hematology, Ankara, Turkey; 2 Gülhane Military Medical Academy, Department of Underwater and Hyperbaric Medicine, Ankara, Turkey; 3 Gülhane Military Medical Academy, Department of Urology, Ankara, Turkey

**Keywords:** Treatment of priapism, Automated red cell exchange, Hyperbaric oxygen therapy, Sickle cell disease

## Abstract

Priapism affects up to 50% of all males with sickle cell disease, and there is no standard treatment. Delayed and unsuccessful treatment leads to corporal fibrosis and impotence. It is therefore necessary to determine the best treatment methods for this complication in order to offer effective interventions to all affected patients. Herein we report an 11-year-old patient with sickle cell disease that presented with priapism 72 h after onset, and was successfully treated with automated red cell exchange and hyperbaric oxygen following unsuccessful surgical and conventional interventions.

## Abstract (tr)

Priapizm orak hücre hastalığı olan tüm erkeklerin yaklaşık yarısında ortaya çıkabilir ve tedavisinde henüz bir fikir birliği oluşmamıştır. Tedavinin başarısız olması ve gecikme korporal fibrozise ve impotansa neden olur. Priapizm gelişen hastalarda en etkili girişimsel yaklaşımı önerebilmek için bu komplikasyonun tedavisinin en iyi şekilde bilinmesine gereklilik vardır. Burada, orak hücre hastalığı olan,72 saattir süregelen priapizm komplikasyonu olan 11 yaşında bir çocuk hastanın cerrahi ve konvansiyonel girişimler yetersiz kaldıktan sonra, otomatik eritrosit değişimi ve hiperbarik oksijen ile başarılı tedavisini bildiriyoruz.

## INTRODUCTION

Priapism is the persistence of an erection in the absence of sexual desire that fails to subside despite orgasm. There are 2 types of priapism: low-flow ischemic (veno-occlusive priapism), which is the form seen in sickle cell disease (SCD), and high-flow priapism (non-ischemic), which is associated with external trauma that damages the cavernosal artery. Sickle cell disease is one of the most common underlying diseases responsible from venous occlusion in low-flow ischemic priapism [1,2]. Priapism occurs in 30%-89% of males with SCD [3,4,5]. The prevalence of priapism in children with SCD was estimated to be 2%-6% [6,7]; however, the prevalence of priapism in children and adolescents with SCD is much higher than previously described [5]. Delayed and unsuccessful treatment leads to corporal fibrosis and impotence. Herein, we report an 11-year-old patient with SCD that presented with priapism 72 h after onset, and was successfully treated with automated red cell exchange and hyperbaric oxygen following unsuccessful surgical and conventional interventions.

## CASE REPORTS

An 11-year-old boy with SCD presented to the emergency department with a painful and persistent erection 72 h after onset. He was known to have SS hemoglobin and had been previously admitted to other hospitals for painful sickle cell crises on 3 occasions: once at the age of 4 years and twice when he was 5 years old. These 3 crises involved upper and lower extremity pain that resolved spontaneously. The diagnosis of homozygous SS hemoglobinopathy was established at the second admission when he presented extremity pain at the age of 5 years.. The patient’s history of recurrent stuttering episodes of priapism was negative. He first presented to another regional hospital with priapism and underwent corporal aspiration in the urology department, which was not successful, and was subsequently referred to our hospital. 

Patient history of trauma, difficulty voiding, dysuria, and hematuria was negative. Causative factors (dehydration, infections, or the use of drugs) of priapism were not noted. Physical examination was unremarkable, except for a swollen, erect penis that was erythematous and very tender. Other symptoms of SCD, including veno-occlusive crisis, acute chest syndrome, acute splenic sequestration, sepsis, and aplastic crisis, were not associated with the priapism. Initial investigations confirmed sickle cell abnormality (92% SS homozygous), based on hemoglobin electrophoresis, and showed severe anemia (Hb: 5.7 g dL^–1^). The patient was treated with hydration, sedation, oxygenation, and transfusion of packed red blood cells. 

As the patient’s priapism had persisted for 72 h, we immediately performed a corpora cavernosa-glans penis shunt. Despite the surgical intervention, detumescence did not occur. The patient was taken into a large, walk-in hyperbaric chamber at 2.5 ATA and 45 ft H_2_O for 90 min session^–1^. Hyperbaric oxygen treatment was initiated. (5 sessions/weekly; totally 11 sessions). On the fourth session of hyperbaric oxygen therapy, erythrocyte apheresis was performed automatically and erythrocyte apheresis was repeated on the fifth session of hyperbaric oxygen therapy. Peripheral access was achieved using a 19-gauge needle. The machine was primed with cross-matched compatible red blood cells. This achieved an Hb level of 10.6 g dL^–1^ with an Hb S fraction of 7.7%. There were no procedural complications. Upon examination after the first erythrocyte aphaeresis, improvement in the degree of erection and pain was evident, and priapism almost completely resolved after the second aphaeresis treatment. Hyperbaric oxygen treatment were applied 11 sessions. The patient’s priapism continued for 6 d before detumescence was complete. We did not observe any complications related to hyperbaric oxygen treatment in our patient. During a 4-year follow up period priapism did not reoccur.

## DISCUSSION

It is thought that priapism is caused by sickle cells occluding the venous outflow of blood from the corpora of the penis. Episodes most commonly start during sleep or upon awakening, last about 2 h, and first occur during adolescence and pre-puberty. Some episodes resolve spontaneously within 2-3 h. Prolonged episodes can cause irreversible ischemic injury and lead to impotence. Symptoms of priapism are classified as acute (lasting >3 h), recurrent or stuttering (lasting ≤3 h and resolving spontaneously), and chronic, which is a rare condition in which the penis is persistently semi-erect, but not painful [8]. Priapism is usually accompanied by pain and tenderness. 

The presented patient was an 11-year-old boy with SCD. Priapism can occur in all age groups, with reported peak incidences in SCD patients between the ages of 5 and 10 years, and 20 and 50 years [2]. Treatment for acute attacks includes hydration, sedation, oxygenation, aspiration, and irrigation with an α-adrenergic agonist and exchange transfusion [9,10,11,12]; these interventions may be helpful within the initial 10-12 h of onset [13]. Surgical treatments for priapism are designed to remove blood from the corpora cavernosa. Early intervention with corporeal aspiration and irrigation with adrenergic agonists was recently reported to be successful in an outpatient clinic [14]. Prior to presentation to our hospital, the presented patient underwent corporal aspiration that was unsuccessful, and the initial therapies; hydration, sedation, oxygenation was not applied.

At presentation to our hospital (72 h after the onset of symptoms) it was too late for conventional treatment approaches in the presented case. A corpora cavernosaglans penis shunt was performed immediately in addition to conventional management with hydration, sedation, and oxygenation, but the symptoms were not relieved. As reported, the success of surgical treatment is associated with its timing (<48 h of the onset of symptoms). Patients suffer physically and most progress to impotency, in part because of delayed treatment [15,16]. The precise mechanism of priapism in patients with SCD remains unclear. It is assumed that a normal erection decreases oxygen tension in the corpora cavernosa, predisposing to erythrocyte sickling [17]. With regard to pathophysiology, hyperbaric oxygen treatment has been successfully used for SCD complications, especially retinopathy, hyphema, and painful crisis [18,19,20,21]. To the best to our knowledge the literature does not contain any data on hyperbaric oxygen treatment for priapism in SCD patients.

In consideration of the presented patient’s serious condition, we initiated hyperbaric oxygen treatment. During this period automated red cell exchange was performed for 2 d and Hb S decreased to 7.7% from 92%, which is a desired level. The benefit of red cell exchange transfusion in SCD patients with priapism remains unclear [22]. McCarthy et al. reported that automated red cell exchange is not useful when initiated ≥1 d after the onset of priapism and when conservative management fails [8]. In contrast, we successfully performed automated red cell exchange 4 d after the onset of priapism, even though previous surgical and conventional treatments failed. Although hyperbaric oxygen appears to have no effect on the morphology of sickle cells in vitro [23], we think it was beneficial in the presented patient. 

Hyperbaric oxygen therapy can result in arterial oxygen tension in the range of 1000-1600 mmHg and tissue oxygen tension close to 400 mmHg. Several indications for the use of hyperbaric oxygen therapy have been described [24], including carbon monoxide poisoning, necrotizing soft tissue infection, decompression sickness, and arterial gas embolism. On the other hand, there are some complications and side effects related to hyperbaric oxygen therapy [25]; pressure equalization problems in the middle ear, sinus squeeze, and tooth squeeze are common complications associated with hyperbaric oxygen therapy. No randomized or controlled studies on the complications of hyperbaric oxygen therapy in pediatric patients have been reported, but a few case reports have been reported. The complications associated with hyperbaric oxygen therapy are strongly correlated with the number of treatments. Ambirou et al. reported a complication rate of 8.1% in patients that received 11-29 treatments, and that when the number of hyperbaric oxygen treatments was ≥30 the complication rate was 17.1% [26]. In the presented patient hyperbaric oxygen treatment was completed after 11 sessions, without any complications. Despite the use of combination treatments, low-flow priapism may result in impotence or erectile dysfunction secondary to corporeal fibrosis [15]. In conclusion, the results obtained in the present case suggest that automated red cell exchange and hyperbaric oxygen may be a good combination for the treatment of resistant priapism in SCD patients. 

## CONFLICT OF INTEREST STATEMENT

The authors of this paper have no conflicts of interest, including specific financial interests, relationships, and/ or affiliations relevant to the subject matter or materials included.
